# Fermented Oyster (*Crassostrea gigas*) Extract Cures and Prevents Prednisolone-Induced Bone Resorption by Activating Osteoblast Differentiation

**DOI:** 10.3390/foods11050678

**Published:** 2022-02-25

**Authors:** Ilandarage Menu Neelaka Molagoda, Athapaththu Mudiyanselage Gihan Kavinda Athapaththu, Eui Kyun Park, Yung Hyun Choi, You-Jin Jeon, Gi-Young Kim

**Affiliations:** 1 Department of Marine Life Science, Jeju National University, Jeju 63243, Republic of Korea; neelakagm2012@gmail.com (I.M.N.M.); gihankavinda@yahoo.com (A.M.G.K.A.); youjinj@jejunu.ac.kr (Y.-J.J.); 2 Research Institute for Basic Sciences, Jeju National University, Jeju 63243, Republic of Korea; 3 Department of Bioprocess Technology, Faculty of Technology, Rajarata University of Sri Lanka, Mihintale 50300, Sri Lanka; 4 Department of Oral Pathology and Regenerative Medicine, School of Dentistry, Kyungpook National University, Daegu 41940, Republic of Korea; epark@knu.ac.kr; 5 Department of Biochemistry, College of Korean Medicine, Dong-Eui University, Busan 47227, Republic of Korea; choiyh@deu.ac.kr

**Keywords:** fermented oyster, prednisolone, osteoblast, osteoclast

## Abstract

Osteoporosis is a bone resorptive disease characterized by the loss of bone density, causing an increase in bone fragility. In our previous study, we demonstrated that gamma aminobutyric acid-enriched fermented oyster (*Crassostrea gigas*) extract (FO) stimulated osteogenesis in MC3T3-E1 preosteoblast cells and vertebral formation in zebrafish. However, the efficacy of FO in prednisolone (PDS)-induced bone resorption remains unclear. In this study, we evaluated the osteogenic potential of FO in MC3T3-E1 preosteoblast cells and zebrafish larvae under both PDS-pretreated and PDS-post-treated conditions. We found that FO recovered osteogenic activity by upregulating osteoblast markers, such as alkaline phosphatase (*ALP*), runt-related transcription factor 2, and osterix, in both PDS-pretreated and post-treated MC3T3-E1 osteoblast cells and zebrafish larvae. In both conditions, PDS-induced decrease in calcification and *ALP* activity was recovered in the presence of FO. Furthermore, vertebral resorption in zebrafish larvae induced by pretreatment and post-treatment with PDS was restored by treatment with FO, along with the recovery of osteogenic markers and downregulation of osteoclastogenic markers. Finally, whether FO disturbs the endocrine system was confirmed according to the Organization for Economic Cooperation and Development guideline 455. We found that FO did not stimulate estrogen response element-luciferase activity or proliferation in MCF7 cells. Additionally, in ovariectomized mice, no change in uterine weight was observed during FO feeding. These results indicate that FO effectively prevents and treats PDS-induced osteoporosis without endocrine disturbances.

## 1. Introduction

Glucocorticoids, which are synthetic adrenal corticosteroids, are considered a common etiology of drug-induced osteoporosis [1]. Glucocorticoids are generally prescribed as anti-inflammatory [2] and analgesic agents [3], which are known to interact with glucocorticoid receptors [4]. The anti-inflammatory properties of glucocorticoids are associated with the inhibition of immune cell function and consequent suppression of the secretion of inflammatory cytokines, such as interleukin (IL)-1β [5]. Nevertheless, clinical investigations have revealed that 20% of the prevalence in osteoporosis patients is attributed to glucocorticoid medication, and the incidents are higher among patients over 65 years of age and who received medication with glucocorticoids over 3-month periods [6]. Direct activity of glucocorticoids on osteoblasts and the bone micro-environment are mediated by the downregulation of the expression and release of bone matrix regulators such as collagen 1 alpha 1 (*Col1α1*) and osteocalcin [7]. In addition, glucocorticoid medication is associated with vitamin D resistance, which consequently decreases calcium absorption from the gastrointestinal tract, causing increased renal excretion of calcium [8,9]. Estrogen or androgen deficiencies as a result of the hypogonadism caused by glucocorticoids also deteriorate the differentiation of mesenchymal stem cells toward the osteoblast precursor cells and prevent the terminal differentiation of osteoblasts, resulting in a decrease in mature osteoblasts [10]. Furthermore, shifting the differentiation of stromal bone marrow cells toward the adipocytic lineage has also been recorded in response to glucocorticoid treatment [11]. This indicates that overexposure to glucocorticoids leads to severe bone resorptive diseases such as osteoporosis in humans.

Prednisolone (PDS), the active form of prednisone, is a glucocorticoid and is basically prescribed as an anti-inflammatory, anti-allergy, and anti-autoimmune disorder agent [12,13]. However, long-term administration of PDS results in fluid and electrolyte disturbances, gastrointestinal problems, endocrine disruptions, metabolic disorders, and bone diseases such as osteoporosis [14]. Among them, PDS-mediated osteoporosis is implicated with increased apoptosis, inhibition of anabolic activities of osteocytes, and decreased secretion of osteoblast matrix, causing osteonecrosis and fractures in bone [15]. Additionally, long-term use of PDS impairs the expression of master osteoblast differentiation regulators, including runt-related transcription factor 2 (*RUNX2*), osterix (*OSX*), and alkaline phosphatase (*ALP*), while increasing the expression of osteoclast activators such as cathepsin K (CTSK), nuclear factor of activated T-cells 1 (NFATc-1), receptor activator of nuclear factor κB (RANK), and acid phosphatase (ACP) [16]. Bisphosphonates are commonly used to treat glucocorticoid-induced osteoporosis and reduce the risk of bone fractures [17]. However, approximately 50–60% people administered with bisphosphonates excrete them from the kidneys, indicating that the efficacy of bisphosphonates is low. Moreover, renal esophageal and acute phase complications have been recorded in the clinical trials medicated by bisphosphonates [18]. Therefore, it is important to identify effective and natural alternatives for the treatment and prevention of osteoporosis caused by glucocorticoids.

Fermented products have recently received potent attention because of their powerful pharmaceutic activities and low side-effects [19,20]. In our previous studies, we demonstrated that fermented oyster *Crassostrea gigas* (FO) extract promoted osteoblast differentiation and bone formation by activating the Wnt/β-catenin signaling pathway [21], concomitant with an increase in growth performance by upregulating insulin-like growth factor-1 (IGF-1) [22]. Additionally, we found that FO prevented ovariectomy (OVX)-induced bone loss [23,24]. Nevertheless, the therapeutic and preventive effects of FO on glucocorticoid-induced bone resorption such as osteoporosis have not been elucidated. In this study, we evaluated the potential of FO under both PDS-pretreated (therapeutic effect) and PDS-post-treated (preventive effect) conditions in preosteoblast MC3T3-E1 cells and zebrafish larvae. Additionally, the safety of FO on estrogen disturbance was tested in accordance with the Organization for Economic Cooperation and Development (OECD) guideline 455 (TG455).

## 2. Materials and Methods

### 2.1. Reagent and Antibody

Calcein, alizarin red, PDS, tricaine methanesulfonate, methylcellulose, β-glycerophosphate (GP), 17β-estradiol (E2), and ICI 182,780 were purchased from Sigma-Aldrich Chemical Co. (St. Louis, MO, USA). A TRACP & *ALP* double-staining kit was purchased from Takara Bio Inc. (Kusatsu, Shiga, Japan). Minimum essential medium alpha modification (α-MEM), fetal bovine serum (FBS), and penicillin–streptomycin solution (100×) were purchased from WELGENE (Gyeongsan, Gyeongsangbukdo, Republic of Korea). Antibodies for *ALP* (sc-398461), *RUNX2* (sc-101145), *OSX* (sc-393325), and β-actin (sc-8432) were purchased from Santa Cruz Biotechnology (Santa Cruz, CA, USA). FO (product name: FO100) was obtained from Marine Bioprocess Co. (Busan, Republic of Korea) [21].

### 2.2. Cell Culture and Osteoblast Differentiation

Mouse MC3T3-E1 preosteoblast cells were purchased from American Type Culture Collection (ATCC, Manassas, VA, USA) and cultured in α-MEM containing 10% FBS and penicillin–streptomycin solution. The cells were seeded at a density of 1 × 10^4^ cells/mL in 6-well plates, and FO was used to evaluate both the therapeutic and preventive effects on PDS-induced bone resorption. For the therapeutic effect, 10 μM PDS was pretreated to MC3T3-E1 cells for two days, followed by treatment with various concentrations of FO (0–200 μg/mL) for five days. To evaluate the preventive effect, FO (0–200 μg/mL) was administered 2 h before 10 μM PDS exposure for seven days. Fresh media were replenished with FO and PDS every two days.

### 2.3. Alizarin Red Staining

To evaluate calcium deposition, MC3T3-E1 cells (1 × 10^4^ cells/mL) were seeded and treated with 10 μM PDS and FO (0–200 μg/mL) for seven days. Afterward, the cells were washed with PBS, fixed with 4% paraformaldehyde for 10 min at 37 °C. Then the fixed cells were stained with 2% alizarin red for 30 min. Images were obtained using phase-contrast microscopy (Macrotech, Goyang, Gyeonggido, Korea).

### 2.4. ALP Staining

MC3T3-E1 cells (1 × 10^4^ cells/mL) were seeded and then treated with 0–200 μg/mL FO and 10 μM PDS. At seven days, *ALP* activity was measured using TRACP & *ALP* double-staining kit (Takara Bio Inc., Kusatsu, Shiga, Japan). According to the manufacturer’s instructions, the cells were rinsed with PBS and fixed for 5 min. Afterward, the *ALP* substrate was added and incubated at 37 °C. The stained cell images were obtained using phase-contrast microscopy (Macrotech).

### 2.5. Reverse Transcription–Polymerase Chain Reaction (RT-PCR) Using MC3T3E-1 Cells

MC3T3-E1 cells were treated with 0–200 μg/mL FO and 10 μM PDS for seven days, and total RNA was extracted using the Easy-BLUE Total RNA Extraction Kit (iNtRON Biotechnology, Sungnam, Gyeonggi, Korea). RNA was reverse-transcribed using MMLV reverse transcriptase (iNtRON Biotechnology), and gene amplification was performed. All mouse primers used in this study are listed in Table 1 [21].

### 2.6. Protein Extraction and Western Blotting

MC3T3-E1 cells were treated with 0–200 μg/mL FO and 10 μM PDS for seven days. Total proteins were extracted using radioimmunoprecipitation assay (RIPA) lysis buffer (iNtRON Biotechnology) with protease inhibitors (Sigma-Aldrich). Bio-Rad Protein Assay Reagent (Bio-Rad, Hercules, CA, USA) was used to quantify the proteins. Equal amounts of protein were separated by SDS-polyacrylamide gel electrophoresis, transferred onto a polyvinylidene fluoride (PVDF) membrane (Thermo Fisher Scientific, Waltham, MA, USA), and then immunoblotted with the indicated antibodies. For the visualization, an Enhanced Chemiluminescence Plus Kit (Thermo Fisher Scientific) was used and the images were obtained using ImageQuant LAS 500 (GE Healthcare Bio-Sciences AB, Uppsala, Sweden). β-Actin was used as the house keeping protein.

### 2.7. Zebrafish Maintenance and Vertebral Staining

All the zebrafish experiments were conducted as described by the standard guidelines of the Animal Care and Use Committee of Jeju National University (Jeju Special Self-Governing Province, Republic of Korea; approval no.: 2021-0066). To evaluate the therapeutic effect of FO, zebrafish larvae at 5 days post fertilization (dpf) were pretreated with 20 μM PDS for two days, followed by treatment with FO (0–100 μg/mL) for another two days in the presence and absence of PDS. For the preventive effect, FO was pretreated for 2 h, followed by treatment with PDS for four days. The media were replenished with FO and PDS every two days. At 9 dpf, vertebrae were visualized using 0.3% calcein green fluorescent staining. After extensive rinsing, the larvae were anesthetized in 0.04% tricaine methanesulfonate solution and mounted on depression slides using 3% methylcellulose. Fluorescent images were obtained using a CELENA S digital imaging system (Logos Biosystems, Anyang, Gyeonggido, Korea).

### 2.8. RT-PCR Using Zebrafish Larvae

Zebrafish larvae at 5 dpf were treated with 0–100 μg/mL FO and 10 μM PDS, and mRNA was extracted using an Easy-BLUE Kit (iNtRON Biotechnology) at 9 dpf. Briefly, RNA were reverse-transcribed using MMLV reverse transcriptase (iNtRON Biotechnology), and PCR was performed using specific primers shown in Table 2 [25,26].

### 2.9. Relative Uterine Weight/Body Weight in OVX Mice

In our previous study [23], OVX-induced osteoporosis mice were prepared according to the guidelines for the care and use of laboratory animals of Kyungpook National University (approval no.: 2017-57). In brief, OVX mice were randomly separated into five groups: sham-operated with vehicle (distilled water, shame), OVX with vehicle (OVX + V), OVX with E2 (OVX + E2), OVX with 100 mg/kg FO, and OVX with 200 mg/kg FO. FO was dissolved in distilled water and orally fed for four weeks. E2 (10 μg/kg) was intraperitoneally administered daily. Upon termination, uteri were quickly removed from the connective tissues, blotted, and weighed. The relative uterine weight was determined by calculating the uterine weight in terms of body weight.

### 2.10. ERE-Luciferase Activity in MCF-7 Cells

MCF-7 cells (ATCC, Manassas, MD, USA) were stably transfected with 1 μg human ERE-luciferase reporter plasmid (Addgene plasmid no. 11354) and 0.2 μg pCMV-β-gal (Addgene plasmid no. 155) using LipofectamineTM 2000 (Invitrogen, Paisley, UK). After 6 h of transfection, the cells were maintained for 24 h in fresh DMEM in the abscense of phenol red (supplemented with 10% charcoal dextran-treated FBS), followed by incubation with FO (2 × 10^−4^–2 × 10^2^ μg/mL), corticosterone (10^−11^–10^−5^ M), genistein (10^−10^–10^−4^ M), and E2 (10^−14^–10^−8^ M). In a parallel experiment, E2 (10^−9^ M) was treated in the presence of ICI 182,780 (10 μM, an ER antagonist) for 24 h. The luciferase and β-galactosidase activities were measured according to the manufacturer’s specifications (Promega, Madison, WI, USA).

### 2.11. Cell Proliferation Assay

The viability was evaluated using a Cellrix Viability Assay Kit (MediFab, Seoul, Republic of Korea) based on water-soluble tetrazolium (WST)-1. Briefly, MCF-7 cells were seeded at a density of 1 × 10^4^ cells/mL for 24 h, followed by treatment with 200 μg/mL FO, 10^−9^ M E2, 10 μM ICI 182,780, or E2 + ICI 182,780 for three days. According to the manufacturer’s specifications, the WST-1 cell proliferation reagent (10 μL) was added to each well, the plates were incubated at 37 °C for 2 h, and the cell viability was quantified at 450 nm using a microplate spectrophotometer (BioTek Instruments Inc., Winooski, VT, USA).

### 2.12. Statistical Analysis

All data represent in this study refer to at least three independent experiments, and the mean ± SEM is indicated. Significant differences were determined using Student’s *t*-test and an unpaired one-way ANOVA test with Bonferroni correction. Statistical significance was set at *** and ^###^ *p* < 0.001, ** *p* < 0.01, and * *p* < 0.05.

## 3. Results

### 3.1. PDS-Induced Anti-Osteogenic Activity Decreases in MC3T3-E1 Cells by Post-Treatment with FO

**Figure 1:**
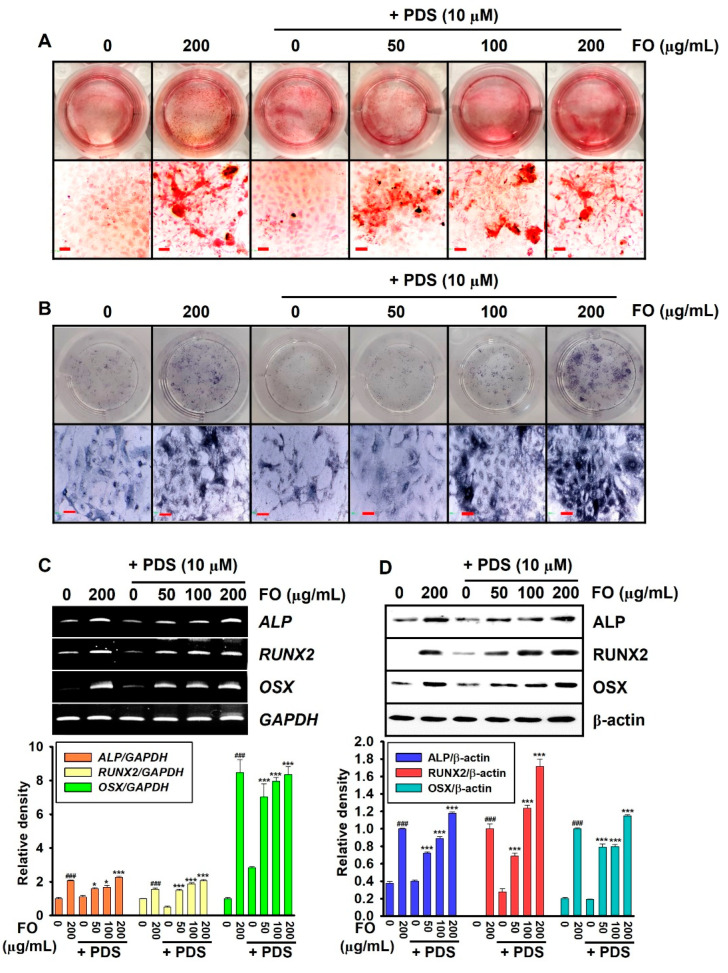
FO promotes osteogenic activity in prednisolone (PDS)-pretreated MC3T3-E1 cells. Mouse preosteoblast MC3T3-E1 cells (1 × 10^4^ cells/mL) were pretreated with 10 μM PDS for two days prior to treatment with FO (0–200 μg/mL) for five days. Fresh media with FO and/or PDS were replenished every two days. At day 7, (**A**) bone mineralization was evaluated by alizarin red staining, and (**B**) *ALP* activity was evaluated using a TRACP & *ALP* Double-Staining Kit. (**C**) In a parallel experiment, total mRNA was extracted, and RT-PCR was performed to evaluate the gene expressions of *ALP*, *RUNX2*, and *OSX*. *GAPDH* was used as the internal control. (**D**) Total proteins were extracted, and Western blotting was performed to evaluate the expression of *ALP*, *RUNX2* and *OSX* proteins. β-Actin was used as the internal control. All data are presented as means ± standard error of the mean (^###^ *p* < 0.001 vs. untreated MC3T3-E1 cells; * *p* < 0.05 and *** *p* < 0.001 vs. PDS-treated MC3T3-E1 cells). *ALP*: alkaline phosphatase; *RUNX2*: runt-related transcription factor 2; *OSX*: osterix; *GAPDH*: glyceraldehyde 3-phosphate dehydrogenase.

To evaluate the therapeutic activity of FO on PDS-induced anti-osteogenic activity in MC3T3-E1 cells, the cells were pretreated with PDS for two days prior to exposure to FO for another five days. As expected, FO itself considerably increased bone mineralization (Figure 1A) and *ALP* activity (Figure 1B) in the absence of PDS. However, PDS conspicuously inhibited the activity of MC3T3-E1 cells. FO also restored the anti-osteogenic activity of PDS-treated MC3T3-E1 cells in a concentration-dependent manner. Furthermore, the osteoblastic marker genes, including *ALP*, *RUNX2*, and *OSX*, were evaluated under PDS-treated conditions on day 7. Pretreatment with PDS significantly lowered the expression of osteoblast marker genes compared to that in the untreated cells, whereas FO restored gene expression in a concentration-dependent manner (Figure 1C). Consistent with the transcriptional expression of the osteoblast marker genes, Western blot analysis revealed that FO remarkably restored PDS-induced inhibition of the expression levels of *ALP*, *RUNX2*, and *OSX* (Figure 1D). These results indicate that FO effectively restores PDS-induced anti-osteogenic activity in MC3T3-E1 cells.

### 3.2. Post-Treatment with FO Overcomes PDS-Induced Delay of Vertebral Formation in Zebrafish Larvae

**Figure 2:**
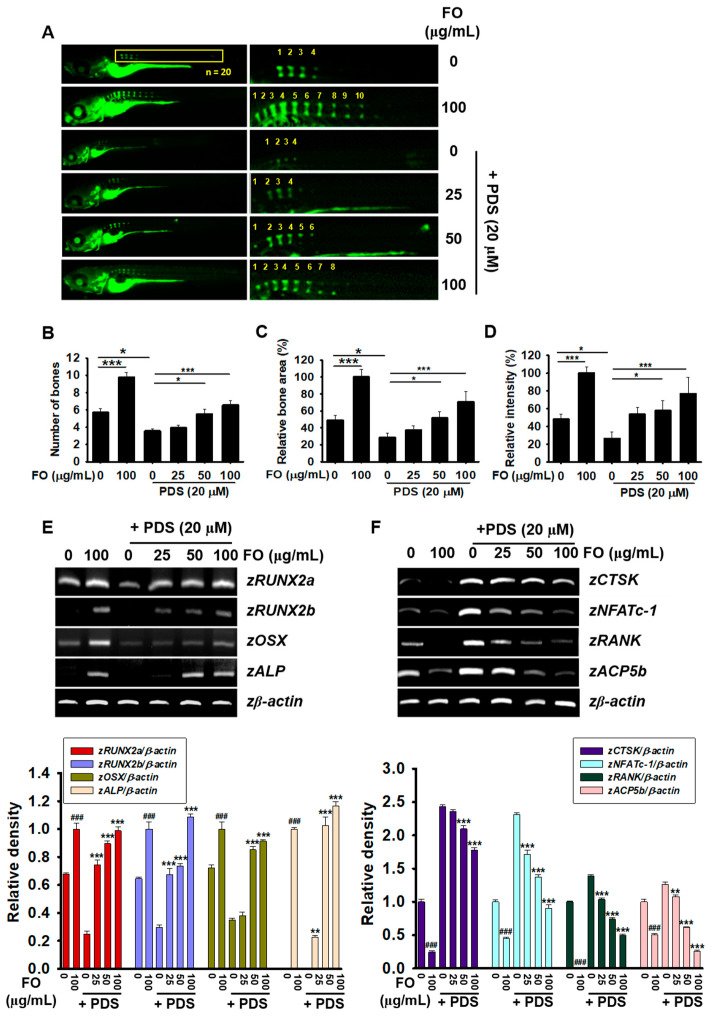
FO prevents PDS-induced bone resorption in zebrafish larvae. Zebrafish larvae (n = 20) at 5 days post fertilization (dpf) were pretreated with 20 μM PDS for two days prior to treatment with FO (0–100 μg/mL) for another two days. Fresh media with FO and/or PDS were replenished at 7 dpf. (**A**) At 9 dpf, zebrafish larvae were stained with 0.03% calcine and observed under fluorescence microscopy. Numbers show vertebrae (right panels). (**B**) Each vertebral number was manually counted and indicated. (**C**) Relative bone area and (**D**) bone intensity were calculated using imageJ software and expressed. All data are presented as means ± standard error of the mean (* *p* < 0.05 and *** *p* < 0.001). In a parallel experiment, total mRNA was extracted, and RT-PCR was performed to evaluate the gene expression of (**E**) osteoblast-related marker genes such as *zRUNX2a*, *zRUNX2b*, *zOSX*, and *zALP* and (**F**) osteoclast-related marker genes such as *zCTSK*, *zNFATc-1*, *zRANK*, and *zACP5b*. *zβ-Actin* was used as the internal control. All data are presented as means ± standard error of the mean (^###^ *p* < 0.001 vs. untreated zebrafish larvae; ** *p* < 0.01, and *** *p* < 0.001 vs. PDS-treated zebrafish larvae). z: zebrafish; *RUNX2a/b*: runt-related transcription factor 2a/b; *OSX*: osterix; *ALP*: alkaline phosphatase; CTSK: cathepsin K; NFATc-1: nuclear factor of activated T-cells; cytoplasmic 1; RANK: receptor activator of nuclear factor κB; and ACP5b: Acid phosphatase 5b.

Zebrafish larvae at 5 dpf were pretreated with PDS for two days prior to treatment with 0–100 μg/mL FO for another two days. As shown in Figure 2A, treatment with 100 μg/mL FO increased vertebral formation in zebrafish larvae at 9 dpf, and in the PDS-pretreated condition, FO restored vertebral formation attenuated by PDS. FO promoted the vertebrae number to 9.80 ± 0.53 in accordance with increased bone area (100.00 ± 8.67%) and relative bone density (100.00 ± 7.18%). However, PDS moderately decreased vertebral number from 5.72 ± 0.42 to 3.56 ± 0.22 (Figure 2B), bone area from 48.80 ± 5.65% to 28.58 ± 5.12% (Figure 2C), and relative bone density (Figure 2D) from 47.95 ± 5.61% to 26.41 ± 7.39%, compared with those in the untreated zebrafish larvae. Furthermore, treatment with FO in the presence of PDS reversed the PDS-induced bone resorption in a dose-dependent manner as 3.93 ± 0.28, 5.53 ± 0.58, and 6.86 ± 0.49 in vertebral number; 37.44 ± 4.95%, 51.59 ± 7.33%, 70.64 ± 12.06% in relative bone area; and 53.46 ± 7.81%, 57.69 ± 11.32%, and 76.90 ± 18.25% in relative bone density at 25, 50, and 100 μg/mL FO, respectively. Additionally, whether FO regulates the expression of osteogenic and osteoclastogenic genes was evaluated in PDS-pretreated zebrafish larvae at 9 dpf. As shown in Figure 2E, the expression of z*RUNX2a*, *zRUNX2b*, *zOSX*, and *zALP* was markedly increased in the presence of 100 μg/mL FO. However, they were completely downregulated in PDS-treated zebrafish larvae. In the PDS-pretreated condition, FO significantly restored the expression of osteogenic marker genes in a dose-dependent manner (Figure 2E). Furthermore, PDS remarkably increased the expression of osteoclastogenic genes, including z*CTSK*, *zNFATc-1*, *zRANK*, and *zACP5b*, in zebrafish larvae at 9 dpf (Figure 2F). However, FO mitigated PDS-induced osteoclastogenic gene expression in a dose-dependent manner. Overall, these results indicate that FO stimulates osteogenesis and inhibits osteoclastogenesis in PDS-pretreated zebrafish larvae, leading to the stimulation of vertebral formation.

### 3.3. Pretreatment with FO Prevents PDS-Induced Anti-Osteogenic Activity in MC3T3-E1 Cells

**Figure 3:**
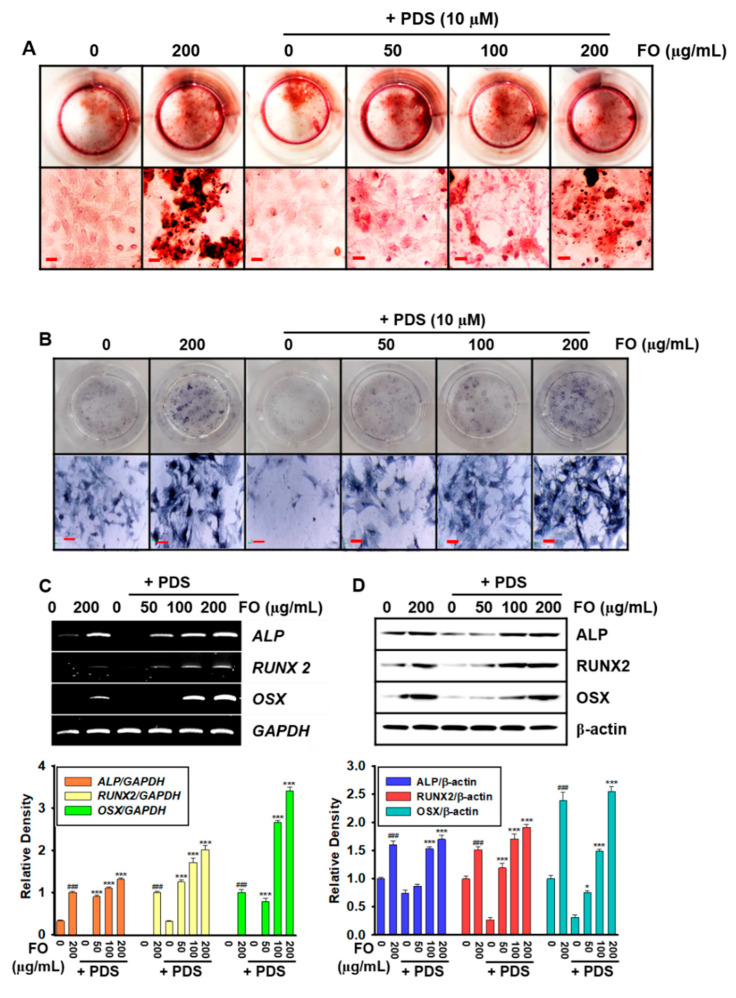
Prednisolone (PDS)-induced anti-osteogenic activity was inhibited by pretreatment with FO in MC3T3-E1 cells. MC3T3-E1 cells (1 × 10^4^ cells/mL) were pretreated with FO (0–200 μg/mL) for 2 h prior to treatment with 10 μM PDS for seven days. Fresh media with FO and/or PDS were replenished every two days. At day 7, (**A**) bone mineralization and (**B**) *ALP* activity were evaluated using alizarin red staining and a TRACP & *ALP* Double-Staining Kit, respectively. (**C**) Total mRNA was extracted, and RT-PCR was performed to evaluate the gene expressions of *ALP*, *RUNX2*, and *OSX*. *GAPDH* was used as the internal control. (**D**) Total proteins were extracted, and Western blotting was performed to evaluate the expression of *ALP*, *RUNX2*, and *OSX*. β-Actin was used as the internal control. All data are presented as means ± standard error of the mean (^###^ *p* < 0.001 vs. untreated MC3T3-E1 cells; * *p* < 0.05, and *** *p* < 0.001 vs. PDS-treated MC3T3-E1 cells). *ALP*: alkaline phosphatase; *RUNX2*: runt-related transcription factor 2; and *OSX*: osterix.

As we confirmed that post-treatment with FO restored osteoblastogenesis and consequent vertebral formation in PDS-pretreated MC3T3-E1 cells and zebrafish larvae, we pretreated the cells with FO for 2 h prior to exposure to 10 μM PDS. Alizarin red staining of bone mineralization (Figure 3A) and *ALP* staining (Figure 3B) revealed that pretreatment with FO dose-dependently prevented the PDS-mediated inhibitory effect on bone mineralization and *ALP* activity. In addition, both RT-PCR (Figure 3C) and Western blot analysis (Figure 3D) revealed that FO, in PDS-pretreated MC3T3-E1 cells, restored the expression of osteogenic markers, including *ALP*, *RUNX2*, and *OSX* in a dose-dependent manner. These results indicated that pretreatment with FO prevented PDS-induced anti-osteoblastic activity.

### 3.4. Pretreatment with FO Prevents PDS-Induced Vertebral Resorption in Zebrafish Larvae

**Figure 4:**
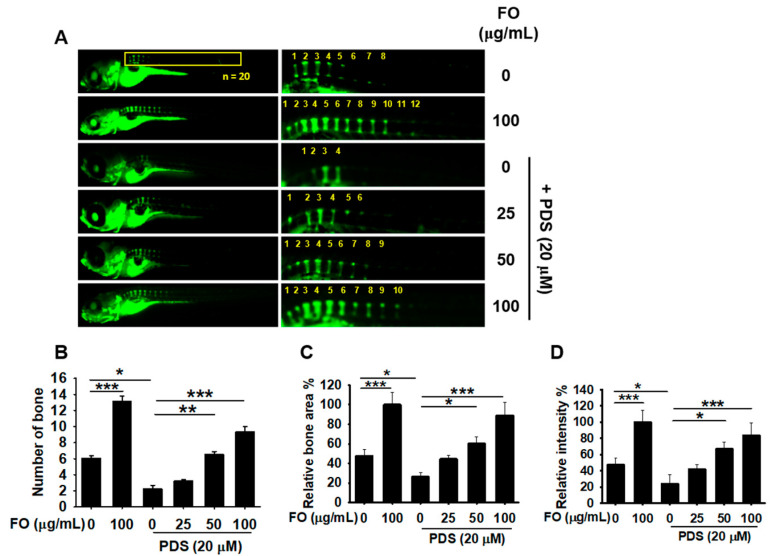

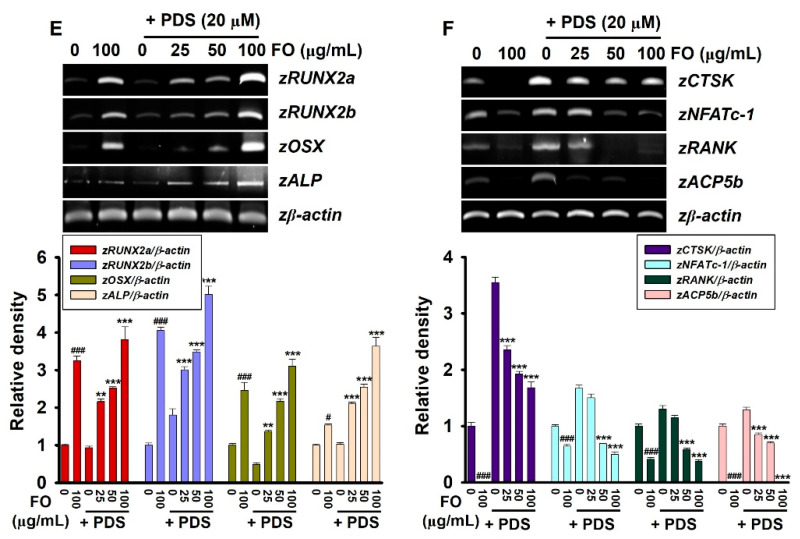
Pretreatment with FO restores vertebral formation in prednisolone (PDS)-post-treated zebrafish larvae. Zebrafish larvae (n = 20) at 5 days post fertilization (dpf) were treated with FO (0–100 μg/mL) for two days, and 20 μM PDS was post-treated for another two days. (**A**) At 9 dpf, zebrafish larvae were stained with 0.03% calcine and observed under fluorescence microscopy. Numbers show vertebrae (right panels) (**B**) Vertebral number was manually counted and indicated. (**C**) Relative bone area and (**D**) bone intensity were calculated using imageJ software and expressed. All data are presented as means ± standard error of the mean (* *p* < 0.05, ** *p* < 0.01, and *** *p* < 0.001). Total mRNA was extracted, and RT-PCR was performed to evaluate the gene expression of (**E**) osteogenic genes, including *zRUNX2a*, *zRUNX2b*, *zOSX*, and *ALP* and (**F**) osteoclastogenic genes such as *zCTSK*, *zNFATc-1*, *zRANK*, and *zACP5b*. *β-Actin* was used as the internal control. All data are presented as means ± standard error of the mean (^#^ *p* < 0.05 and ^###^ *p* < 0.001 vs. untreated zebrafish larvae; ** *p* < 0.01, and *** *p* < 0.001 vs. PDS-treated zebrafish larvae). z: zebrafish; *RUNX2a/b*: runt-related transcription factor 2a/b; *OSX*: osterix; *ALP*: alkaline phosphatase; CTSK: cathepsin K; NFATc-1: nuclear factor of activated T-cells; cytoplasmic 1; RANK: receptor activator of nuclear factor κB; and ACP5b: acid phosphatase 5b.

To evaluate the preventive effect of FO on PDS-induced bone resorption, FO was treated in 5 dpf zebrafish larvae for 2 h prior to exposure to PDS to 9 dpf (Figure 4A). Treatment with 100 μg/mL FO increased vertebral number from 6.06 ± 0.34 to 13.11 ± 0.69 (Figure 4A,B) accompanied with the increased bone area (100.00 ± 12.24%, Figure 4C) and relative bone density (100.00 ± 14.67%, Figure 4D) compared with those in untreated zebrafish larvae. As expected, treatment with PDS significantly inhibited vertebral number (2.32 ± 0.46), relative bone area (28.46 ± 9.81%), and relative bone density (26.99 ± 10.81%). However, pretreatment with FO enhanced vertebral number (3.20 ± 0.24, 6.85 ± 0.37, and 9.31 ± 0.72 at 25, 50 and 100 μg/mL), relative bone area (44.56 ± 3.91%, 60.15 ± 7.01%, and 90.11 ± 13.39% at 25, 50 and 100 μg/mL), and relative bone density (41.87 ± 5.23%, 66.96 ± 8.48%, and 83.86 ± 15.33% at 25, 50 and 100 μg/mL), indicating that FO potently prevents PDS-induced bone resorption. Consistent with vertebral formation data, FO upregulated the expression of osteogenic genes, including z*RUNX2a*, z*RUNX2b*, z*OSX*, and z*ALP* in PDS-treated zebrafish larvae (Figure 4E). In addition, the expression of the PDS-induced osteoclastogenic genes, including z*CTSK*, *zNFACTc-1*, *zRANK*, and *zACP5b*, was downregulated by pretreatment with FO in a dose-dependent manner. These data indicate that pretreatment with FO effectively prevents PDS-induced osteoclastogenic activity and bone resorption in zebrafish larvae.

### 3.5. Estrogenic Activity Is Not Associated with FO

**Figure 5:**
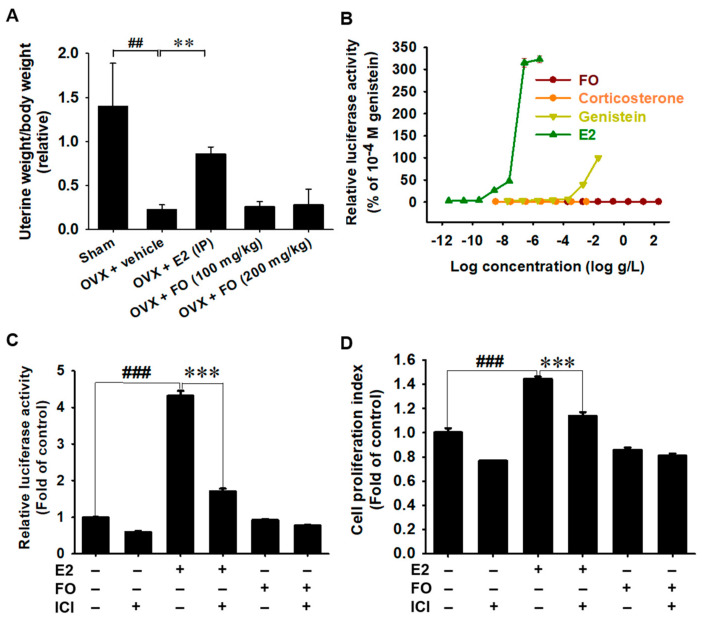
FO is not related to estrogenic activity in ovariectomy (OVX)-induced mice and estrogen response element (ERE)-luciferase transfected MCF-7 cells. (**A**) OVX-induced mice were orally fed with 100 mg/kg and 200 mg/kg FO and intraperitoneally (IP) injected with 10 μg/kg 17β-estradiol (E2) for 4 weeks. After termination, uterine weight was determined by calculating uterine weight in body weight. (**B**–**D**) MCF-7 breast cancer cells (1 × 10^4^ cells/mL) transfected with human estrogen response element (ERE)-luciferase reporter plasmid for 24 h and (**B**) concentration–response curve of transcriptional activation for FO (2 × 10^−4^–2 × 10^2^ μg/mL), corticosterone (10^−11^–10^−5^ M), genistein (10^−10^–10^−4^ M), and E2 (10^−14^–10^−8^ M) were measured using luciferase activity. (**C**,**D**) In a parallel experiment, FO (200 μg/mL) and E2 (10^−9^ M) were treated in the presence or absence of 10 μM ICI 182,780 (ICI), and (**C**) relative luciferase activity and (**D**) relative cell proliferation were calculated. All data are presented as means ± standard error of the mean (and ^###^ *p* < 0.001 and ^##^ *p* < 0.01 vs. untreated MCF-7 cells; *** *p* < 0.001 and ** *p* < 0.01 vs E2-treated MCF-7 cells).

In a previous study, we confirmed that FO prevented OVX-induced bone resorption [24]. Thus, to estimate the safety of FO on the potential estrogenic activity, relative uterine weight/body weight was measured in OVX mice after administering FO for four weeks. A significant decrease in uterine weight/body weight was observed in the OVX mice (Figure 5A). Intraperitoneal injection of E2 significantly increased the relative uterine weight/body weight in OVX mice. However, oral administration of FO did not influence relative uterine weight. According to the ERE-luciferase assay in ER-responsive breast cancer MCF-7 cells, no significant luciferase activity was observed at the concentrations of FO from 2 × 10^−4^ to 2 × 10^2^ μg/mL. However, E2 slightly increased ERE-luciferase activity from 10^−11^ M and to the maximum activity at 10^−8^ M (Figure 5B), which indicates that FO stimulates bone formation in an estrogen-independent manner. Genistein, a well-known phytoestrogen, also caused significant ERE-luciferase activation at 10^−6^ and 10^−5^ M. As expected, corticosterone (10^−11^ to 10^−4^ M) used as a negative control did not affect ERE-luciferase activity. Furthermore, to evaluate whether FO possesses an ER agonist effect, the ERE-luciferase reporter gene construct was transfected into MCF-7 cells, and the activity was measured. As depicted in Figure 5C, E2 significantly increased ERE-luciferase activity (4.32 ± 0.14) compared with that in the untreated MCF-7 cells (0.97 ± 0.02); however, in the presence of ICI 182,780, the activity strongly decreased to 1.70 ± 0.08. Meanwhile, no significant difference in the activity was observed in FO-treated cells (0.92 ± 0.03). Additionally, E2 significantly increased the proliferation of MCF-7 cell from 1.00 ± 0.04 to 1.44 ± 0.02 compared with that of the untreated MCF-7 cells; however, in the presence of ICI, E2-induced cell proliferation decreased to 1.14 ± 0.03 (Figure 5D). Consistent with the luciferase activity data, FO did not cause significant changes in cell proliferation either in the absence (0.85 ± 0.02) or presence (0.81 ± 0.02) of ICI. Overall, these results indicate that FO does not affect the estrogenic activity.

## 4. Discussion

Synthetic adrenal glucocorticoids such as PDS are prescribed as anti-inflammatory and immunosuppressive agents; however, their long-term administration is considered the main etiology of secondary osteoporosis [12,13]. Currently, anti-bone resorptive agents such as bisphosphonates are successfully employed as treatment options. However, low drug efficacy with several off-target effects, including renal toxicity, esophageal ulcers, and acute phase reactions, have been reported [18]. Therefore, natural anabolic agents have gained extensive attention owing to their high efficacy and minimal side effects [7,19]. In our previous studies [21,22], we demonstrated that FO is a potent bone anabolic agent that promotes osteoblast differentiation, bone formation, and growth performance through the Wnt/β-catenin-mediated IGF-1 signaling pathway. In addition, we reported that FO significantly prevented ovariectomy-induced bone resorption by inhibiting osteoclast differentiation and activity [23,24]. The ultra-performance liquid chromatography (UPLC) for amino acids of FO revealed that FO contained approximately 24.5% GABA from the total amino acid content [21]. Furthermore, in our recent studies, we found that GABA directly regulated growth performances in zebrafish larvae through GABA_A_ and GABA_B_ receptors, indicating the potent influence of GABA in FO-mediated growth performances [27]. Nevertheless, the therapeutic and preventive effects of FO have not been elucidated in glucocorticoid-induced secondary bone resorption. In this study, we demonstrated that FO effectively cured and prevented PDS-induced anti-osteogenic activity and bone resorption in MC3T3-E1 and zebrafish larvae.

Osteoblast differentiation is tightly regulated by key transcription factors, including *RUNX2* and *OSX* [28,29]. He et al. [30] discovered that PDS downregulated the transcription of *RUNX2* and *OSX* in zebrafish larvae and induced bone resorption. In addition, the downregulation of *RUNX2* and *OSX* transcriptional activities has been found in PDS-treated mice concomitant with decreased bone area and thickness [16], confirming that PDS has a negative impact on osteoblast differentiation. In our previous study [31], direct action of PDS was also found to downregulate the gene expression of *OCN*, *ALP*, and *Col1α1* in both mouse preosteoblast MC3T3-E1 and zebrafish larvae, impairing the synthesis of extracellular matrix components. Furthermore, PDS remarkably stimulates the expression of matrix metalloproteases (MMPs), including MMP-2, MMP-9, and MMP-13 in mice [32], which subsequently degrades all types of extracellular matrix (ECM) proteins, suggesting that the anti-osteoblast activities of PDS may also be linked to the degradation of ECM components. Furthermore, osteoclastogenesis was previously identified in response to PDS treatment in both zebrafish and mouse models, accompanied by specific expression of osteoclast-specific genes [16,30,31]. Interestingly, we found that FO, under both PDS-pretreated and post-treated conditions, cured and prevented PDS-mediated bone mineralization and resorption in a concentration-dependent manner. Nevertheless, whether FO directly influences the formation of ECM components remains unknown. Although FO has therapeutic and preventive effects on PDS-induced bone resorption, the efficacy of FO should be evaluated in glucocorticoid-mediated osteoporosis at the clinical level.

Endocrine-disrupting compounds (EDCs) such as bisphenol A and 4-nonylphenol have been reported in marine invertebrates and fish [33,34]. Le Curieux-Belfond et al. [35] demonstrated the short-term bioaccumulation of E2 in the pacific oyster, *C*. *gigas*, suggesting that EDCs have adverse effects on differentiation, development, proliferation, and reproduction through endocrine disturbance. In this regard, excessive consumption of oysters and transmission of EDCs may also disrupt the endocrine system in humans. Bateman et al. [36] demonstrated that EDCs led to decreased osteoblast differentiation by targeting *RUNX2* and other osteoblastic marker genes and increased adipogenic differentiation. Therefore, it is essential to prove the absence of EDCs in food resources prior to consumption. OECD guideline 455 (TG455) has recently imposed standard guidelines for the screening and testing of potential EDCs [37]. In our ovariectomy-induced osteoporosis model, E2 restored relative uterine weight. However, FO did not influence the weight, which indicates that FO promotes osteoblast differentiation and vertebral formation regardless of endocrine disturbances. Additionally, E2 induced ERE-luciferase activity in estrogen-responsive MCF-7 breast cancer cells and increased proliferation, but not in FO-treated MCF-7 cells. Previously, we also reported that FO did not directly bind to estrogen receptors and androgen receptors and did not show any changes in the weight of the androgen- and estrogen-dependent organs [31]. Overall, these results indicate that FO promotes osteoblast differentiation and vertebral formation regardless endocrine disturbance.

In summary, our results revealed the therapeutic and preventive effects of FO in PDS-induced osteoporosis. Our findings show that FO may be a potent pharmacological food source to downregulate bone-resorptive diseases such as osteoporosis, by activating osteoblast differentiation and bone formation.

## 5. Conclusions

This study demonstrated the therapeutic and preventive effects of FO on PDS-induced bone resorption. We suggest that FO may be a promising candidate for the treatment and prevention of osteoporosis. Nevertheless, further evaluation through human clinical tests is required to determine whether FO has anti-osteoporotic effects. With respect to its potential as a new natural bone anabolic agent with high efficacy and minimal side effects, it is necessary to verify the effect of FO in clinical trials. In addition, FO did not affect estrogen activity and endocrine disturbance during bone formation, so it can be used as an excellent safeguard for adolescents and women.

## Figures and Tables

**Table 1:** Mouse primers and PCR conditions used in this study.

Gene *	Primer Sequence (5′-3′)	Size	T_m_
*ALP*	F: 5′-TTGTGGCCCTCTCCAAGACA-3′	198 bp	60 °C
	R: 5′-GACTTCCCAGCATCCTTGGC-3′		
*RUNX2*	F: 5′-CATGGTGGAGATCATCGCGG-3′ R: 5′-GGCCATGACGGTAACCACAG-3′	171 bp	60 °C
*OSX*	F: 5′-AAGGCGGTTGGCAATAGTGG-3′ R: 5′-GCAGCTGTGAATGGGCTTCT-3′	194 bp	60 °C
*GAPDH*	F: 5′-ACCACAGTCCATGCCATCAC-3′	480 bp	63 °C
	R: 5′-CACCACCCTGTTGCTGTAGC-3′		

* ALP, alkaline phosphatase; *RUNX2*, runt-related transcription factor 2; *OSX*, osterix; *GAPDH*, glyceraldehyde 3-phosphate dehydrogenase; F, forward; R, reverse; bp, base pairs; T_m_, melting temperature.

**Table 2:** Zebrafish primers and PCR conditions used in this experiment.

Gene *	Primer Sequence (5′-3′)	Size	T_m_
*zRUNX2a*	F: 5′-GACGGTGGTGACGGTAATGG-3′	173 bp	58 °C
	R: 5′-TGCGGTGGGTTCGTGAATA-3′		
*zRUNX2b*	F: 5′-CGGCTCCTACCAGTTCTCCA-3′	149 bp	59 °C
	R: 5′-CCATCTCCCTCCACTCCTCC-3′		
*zOSX*	F: 5′-GGCTATGCTAACTGCGACCTG-3′	153 bp	56 °C
	R: 5′-GCTTTCATTGCGTCCGTTTT-3′		
*zALP*	F: 5′-CAAGAACTCAACAAGAAC-3′	170 bp	48 °C
	R: 5′-TGAGCATTGGTGTTATAC-3′		
*zCTSK*	F: 5′-GGACTCAATCACTATCACT-3′	117 bp	56 °C
	R: 5′-AGAACAAGACATCTAAGACA-3′		
*zNFATc-1*	F: 5′-AACCTTCCTCGTTCCCTCAA-3′	152 bp	57 °C
	R: 5′-CGCTGTTATCCTCCACCTCA-3′		
*zRANK*	F: 5′-GCACGGTTATTGTTGTTA-3′	109 bp	49 °C
	R: 5′-TATTCAGAGGTGGTGTTAT-3′		
*zACP5b*	F: 5′-GCTGCTGCTAACAAACAAT-3′	76 bp	52 °C
	R: 5′-GACCAACCACGATGACAA-3′		
*zβ-actin*	F: 5′-CGAGCGTGGCTACAGCTTCA-3′	155 bp	60 °C
	R: 5′-GACCGTCAGGCAGCTCATAG-3′		

* *zRUNX2a/b*, runt-related transcription factor 2a/b; *zOSX*, osterix; *zALP*, alkaline phosphatase; *zCTSK*, cathepsinK; *zNFATc-1*, nuclear factor of activated T-cells cytoplasmic 1; *zRANK*, receptor activator of nuclear factor κB; *zACP5b*, acid phosphatase 5b; F, forward; R, reverse; bp, base pairs; T_m_, melting temperature.

## Data Availability

The data used to support the findings of this study are available from the corresponding author upon request.
