# Correction: Molagoda et al. Fermented Oyster (*Crassostrea gigas*) Extract Cures and Prevents Prednisolone-Induced Bone Resorption by Activating Osteoblast Differentiation. *Foods* 2022, *11*, 678

**DOI:** 10.3390/foods14234081

**Published:** 2025-11-28

**Authors:** Ilandarage Menu Neelaka Molagoda, Athapaththu Mudiyanselage Gihan Kavinda Athapaththu, Eui Kyun Park, Yung Hyun Choi, You-Jin Jeon, Gi-Young Kim

**Affiliations:** 1 Department of Marine Life Science, Jeju National University, Jeju 63243, Republic of Korea; neelakagm2012@gmail.com (I.M.N.M.); gihankavinda@yahoo.com (A.M.G.K.A.); youjinj@jejunu.ac.kr (Y.-J.J.); 2 Research Institute for Basic Sciences, Jeju National University, Jeju 63243, Republic of Korea; 3 Department of Bioprocess Technology, Faculty of Technology, Rajarata University of Sri Lanka, Mihintale 50300, Sri Lanka; 4 Department of Oral Pathology and Regenerative Medicine, School of Dentistry, Kyungpook National University, Daegu 41940, Republic of Korea; epark@knu.ac.kr; 5 Department of Biochemistry, College of Korean Medicine, Dong-Eui University, Busan 47227, Republic of Korea; choiyh@deu.ac.kr

In the original publication [1], there was a mistake in Figure 3A. This data error occurred due to an unforeseen mistake during the data organization process. The corrected Figure 3 appears below. The authors state that the scientific conclusions are unaffected. This correction was approved by the Academic Editor. The original publication has also been updated.

## Figures and Tables

**Figure 3:**
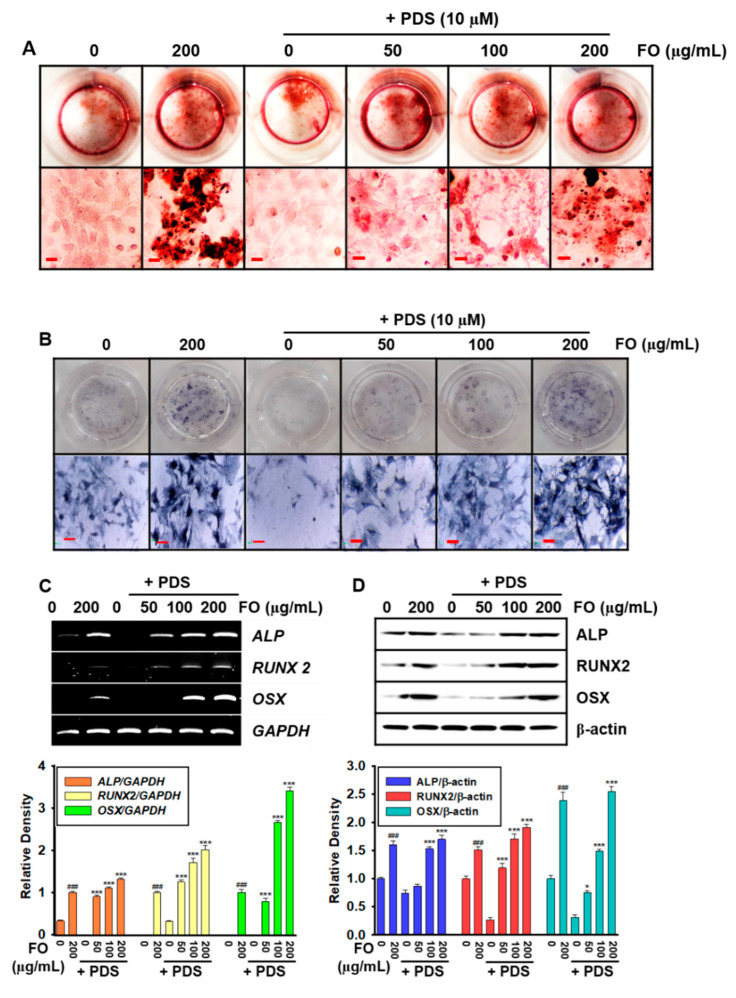
Prednisolone (PDS)-induced anti-osteogenic activity was inhibited by pretreatment with FO in MC3T3-E1 cells. MC3T3-E1 cells (1 × 10^4^ cells/mL) were pretreated with FO (0–200 μg/mL) for 2 h prior to treatment with 10 μM PDS for seven days. Fresh media with FO and/or PDS were replenished every two days. At day 7, (**A**) bone mineralization and (**B**) *ALP* activity were evaluated using alizarin red staining and a TRACP & *ALP* Double-Staining Kit, respectively. (**C**) Total mRNA was extracted, and RT-PCR was performed to evaluate the gene expressions of *ALP*, *RUNX2*, and *OSX*. *GAPDH* was used as the internal control. (**D**) Total proteins were extracted, and Western blotting was performed to evaluate the expression of *ALP*, *RUNX2*, and *OSX*. β-Actin was used as the internal control. All data are presented as means ± standard error of the mean (^###^ *p* < 0.001 vs. untreated MC3T3-E1 cells; * *p* < 0.05, and *** *p* < 0.001 vs. PDS-treated MC3T3-E1 cells). *ALP*: alkaline phosphatase; *RUNX2*: runt-related transcription factor 2; and *OSX*: osterix.
